# The Tonoplast Intrinsic Protein Gene *KvTIP3* is Responsive to Different Abiotic Stresses in *Kosteletzkya virginica*

**DOI:** 10.1155/2020/2895795

**Published:** 2020-01-02

**Authors:** Xiaohua Liu, Jieshan Cheng, Fudong Jiang, Meixia Liang, Junjie Han, Juan Zhang, Hongxia Zhang, Xiaoli Tang

**Affiliations:** ^1^College of Agriculture, Ludong University, 186 Hongqizhong Road, Yantai, Shandong, China; ^2^Key Laboratory of Molecular Module-Based Breeding of High Yield and Abiotic Resistant Plants in Universities of Shandong (Ludong University), 186 Hongqizhong Road, Yantai, Shandong, China; ^3^Yantai Academy of Agricultural Sciences, 26 West Gangcheng Street, Yantai, Shandong, China; ^4^Institute for Advanced Study of Coastal Ecology, Ludong University, 186 Hongqizhong Road, Yantai, Shandong, China

## Abstract

In higher plants, aquaporin proteins (AQPs) play important roles in the uptake of water across cell membranes. However, their functions in halophytes are still largely unknown. In this work, we isolated, cloned, and identified *KvTIP3*, a tonoplast intrinsic protein gene from *Kosteletzkya virginica*. Bioinformatic analyses demonstrated that *KvTIP3* encoded a tonoplast protein with the common properties of AQPs. Further multiple sequence alignment and phylogenetic analyses showed that KvTIP3 shared 65%–82% homology with other AQPs from *Arabidopsis*, cotton, polar, and cocoa. Quantitative real-time PCR (qPCR) analyses revealed that *KvTIP3* was ubiquitously expressed in various tissues such as leaves, stems, and roots, with a predominant expression in roots. In addition, *KvTIP3* transcript was strongly induced by NaCl, low temperature, and ABA in *K. virginica*. Our findings suggest that *KvTIP3* encodes a new AQP possibly involved in multiple abiotic stress responses in *K. virginica*, and *KvTIP3* could be used as a potential candidate gene for the improvement of plants resistant to various abiotic stresses.

## 1. Introduction

Water is one of the essential requirements for all the organisms living on Earth [1, 2]. As a principal member of the biological kingdom, plants have evolved a series of mechanisms to absorb, transport, and utilize water. Unlike animals, plants cannot escape away from the adverse environment, such as drought and salt stress. Therefore, they must ensure the balance of water at both cellular and whole plant levels. AQPs are a family of channel proteins existing in all living organisms and play crucial roles in regulating the movement of water across the cell membrane [3]. For example, 13 AQPs were identified in human genome [4]. 35 and 36 AQPs were identified in *Arabidopsis* and maize, respectively [5, 6]. In addition, the number of AQPs isolated was 28 in *Vitis vinifera* L. [7], 28 in *Beta vulgaris* [8], 55 in *Populus trichocarpa* [9], 41 in *Solanum lycopersicum* [10], 63 in bamboo [11], 66 in *Glycine max* [12], and 71 in cotton [13].

AQPs are integral membrane proteins working in the form of tetramers and each monomer acts as an independent water channel [14]. According to the phylogenetic positions and subcellular localizations, plant AQPs are subdivided into four groups: plasma membrane intrinsic proteins (PIPs), tonoplast intrinsic proteins (TIPs), nodulin 26-like intrinsic proteins (NIPs), and small basic intrinsic proteins (SIPs) [15]. PIPs and TIPs are the most abundant proteins located in the plasma membrane and tonoplast, respectively [15]. Recently, three more groups, uncharacterized intrinsic proteins (XIPs), GlpF-like intrinsic proteins (GIPs), and hybrid intrinsic proteins (HIPs), were also proposed [16, 17]. Therefore, AQPs consist of seven subfamilies. TIPs are AQPs predominantly located at the tonoplast [15]. Structurally, TIPs possess the conserved domains and motifs of AQPs, the six transmembrane helices with N-terminus and C-terminus located in the cytosol, and two NPA (asparagine-proline-alanine) motifs [7, 12]. The transmembrane helices constitute the channel for water and other substrate molecules. The conserved NPA motifs determine the substrate specificity [18]. In addition to transporting water across the membrane, TIPs can also facilitate the transport of other small molecules such as CO_2_, glycerol, NH_3_, arsenite, and silicon [19, 20]. Therefore, TIPs not only play a key role in water uptake but also function in many other aspects during the growth and development of plant.

Recently, an increasing number of researches revealed that TIPs played important functions in plant response to abiotic stresses. In *Arabidopsis*, TIP3;1, TIP3;2, and TIP4;1 were found to be involved in seed dormancy and germination in response to water stress [21]. In the halophyte *Thellungiella salsuginea* (salt cress), *TsTIP1;2* was involved in multiple stresses responses [22]. Recently, AQPs were reported to be involved in abiotic stress response in the halophyte *Eutrema salsugineum* [23]. In *Mesembryanthemum crystallinum*, osmotic stress can induce the relocation of McTIP1;2 from the tonoplast to other membranes [24]. Consistently, expression of *CsTIP2;1* from citrus in tobacco improved the growth and antioxidant capacity of transgenic plants under stress conditions [25]. Similarly, expression of tomato *SlTIP2;2* and jojoba *ScPIP1* enhanced the tolerance of transgenic plants to various abiotic stresses [26]. However, the functions of TIPs are multifaceted. The same environmental stimulus can lead to both upregulated and downregulated expression of different TIPs. Similarly, overexpression of TIPs does not always cause a positive effect on transgenic plants. For example, expression of *GsTIP2;1*, which was responsive to abiotic stresses, depressed salt and dehydration resistance in *Arabidopsis* [27]. Therefore, AQPs could function in various environmental stress responses.

*K. virginica*, a typical halophyte, is a new species with fine salt-tolerant characteristics at both physiological and molecular levels [28]. As an extremophile plant, *K. virginica* is very tolerant to adverse environmental stresses. It could grow and reproduce successfully in soil containing 0.3 to 2.5% sodium [29]. Previously, we examined the global gene expression profiles of *K. virginica* in response to salt stress [28]. Many gene expressions and pathways showed discrepancy. In this work, we isolated and identified a tonoplast aquaporin encoding gene *KvTIP3* from *K. virginica*, which showed the most significant gene expression variation as exhibited by transcriptomic analyses. Our results demonstrate that *KvTIP3* is a typical TIP encoding gene with a responsive expression to different abiotic stresses.

## 2. Materials and Methods

### 2.1. Plant Materials

*K. virginica* seeds were collected from the Yellow River Delta, Shandong Province, China. Seeds were surface sterilized and seedlings were cultivated as described before [30]. Two-week-old seedlings with the same morphology were collected and used for the following experiments as described previously [31, 32].

### 2.2. Abiotic Stress Treatments

Two-week-old seedlings were subjected to high temperature, low temperature, high salinity, osmotic stress, and ABA treatments as described previously [30, 33].

### 2.3. RNA Isolation and cDNA Synthesis

*Total* RNA was extracted and analyzed as described previously [30]. The RNA samples were used to synthesize cDNA with TransScript One-Step gDNA Removal and cDNA Synthesis SuperMix (Transgen, China). Primers used for cDNA synthesis were random primer and anchored oligo (dT)_18_. Reaction was carried out by incubating the reaction mixture at 42°C for 30 min, and then at 85°C for 5 min. Finally, cDNA was examined with Agilent 2100 BioAnalyzer to confirm its quality and quantity.

### 2.4. Cloning and Bioinformatic Analyses of KvTIP3

The full length of *KvTIP3* gene was amplified by RACE technique according to the user's manual (SMART^™^ RACE cDNA Amplification Kit). Gene-specific primers (GSP-F: 5′-TAGTAAACCG CACGGACCACAGA-3′ and GSP-R: 5′-CCAACATCTTAGTAGGCGGACCATT -3′) and nested primers (NGSP-F: 5′-TTCCTCCAAGCAAGGCACCGAAG-3′ and NGSP-R: 5′-GCCTTGGTAGGTTGGAGGTGGGA-3′) were designed according to the sequence of cDNA fragments in our previous transcriptomic sequencing data, which was deposited in Transcriptome Shotgun Assembly (TSA) Sequence Database with the accession number-GCJL00000000 [29]. The obtained gene fragment was ligated into the pEASY-T5 Zero Cloning Vector (Transgen, China) after purification and sequencing.

### 2.5. Quantitative Real-Time RT-PCR (qPCR) Analyses

qPCR was carried out with the instrument ABI PrismSYMBOL 0 \f “Times New Roman” \s 127500 FAST (Applied Biosystems, Foster City, CA). The reaction system was performed using the SYBR Green Real-Time Selected Master Mix (Applied Biosystems by Life technologies), and the reaction condition was performed following the user's manual. cDNA was diluted to the required concentration (<50 ng/*μ*L), and gene-specific primers were designed with the Beacon Designer (BD) 7.0 software based on the RACE results. Gene-specific primers used for qPCR are 5′-ATGCCAACTCGTAGATAC-3′ and 5′-CTAGTAATCTTCAGGAG C-3′. The reaction volume was 20 *μ*L with a composition of 2 *μ*L diluted cDNA template, 10 *μ*L 2 × SYBR Master Mix, 1.5 *μ*L primers, and 6.5 *μ*L double distilled water. A reference gene, *18SrRNA*, which was the most stably expressed gene among the four selected reference genes (*18SrRNA*, *ACT*, *TUA*, and *EF*) under salt stress condition, was used as an internal control [34]. qPCR amplification was performed as following: initial denaturation at 95°C for 2 min, 40 cycles of 15 s at 95°C for denaturation, and 1 min at 60°C for annealing and extension. Fluorescence signal was conducted at the temperature between 60°C and 90°C. The MIQE guidelines: minimum information for publication of quantitative real-time PCR experiment, was strictly followed throughout our experiment [35]. In addition, to guarantee the accuracy of the experiment, the primer specificity of qPCR was insured by the typical melting curve, amplification plot, and the single product of RT-PCR. cDNA was diluted to suitable concentrations to guarantee the Cq values in the optimum range. For all the experiments, each sample was performed in triplicates and three biological replicates were performed.

### 2.6. Data Analysis

All the data were mean values ± SD of three experiments. For qPCR analysis, the ΔΔCq method was used. ANOVA was applied to analyze the significant differences, and the *P* value was kept below 0.05. Sigma Plot 12.0 was used for plotting.

## 3. Results

### 3.1. Isolation and Sequence Analysis of KvTIP3

To understand the possible function of AQPs in abiotic stress tolerance in halophyte, a *K. virginica* homolog of tonoplast intrinsic protein gene, *KvTIP3*, was isolated (GenBank accession no. KT732779). The 1084-bp *KvTIP3* consists of a 154-bp 5′ untranslated region (UTR), a 768-bp encoding region, and a 161-bp 3′ UTR, encoding a 256 amino acid protein (KvTIP3) with a calculated molecular mass of 27 kD and isoelectric point of 6.58, as predicted by ProtParam (http://www.expasy.org/tools/).

### 3.2. Bioinformatic Analysis of KvTIP3

**Figure 1:**
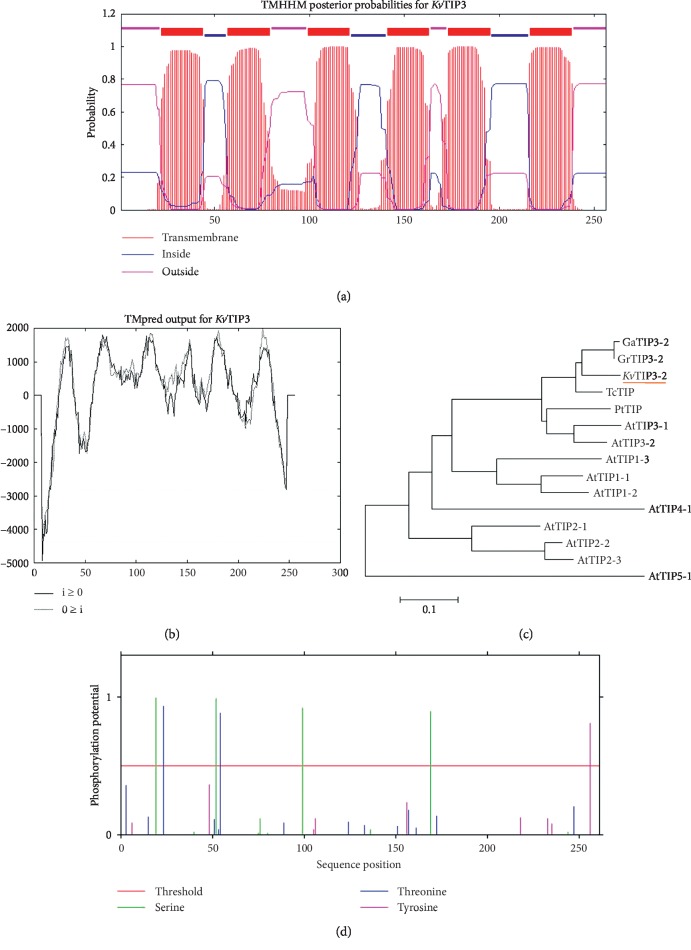
The secondary structure, phosphorylation sites, and phylogenetic analysis of KvTIP3. (a, b) Transmembrane domain analysis of KvTIP3 with TMHHM and TMPred. (c) Phylogenetic analyses of KvTIP3 with other closely related TIPs proteins. All the sequences were extracted from the NCBI database. The phylogenetic tree was constructed by Mega 7.0 program using the neighbor-joining (NJ) method. (d) The potential phosphorylation sites of KvTIP3 predicted with NetPhos 2.0.

**Figure 2:**
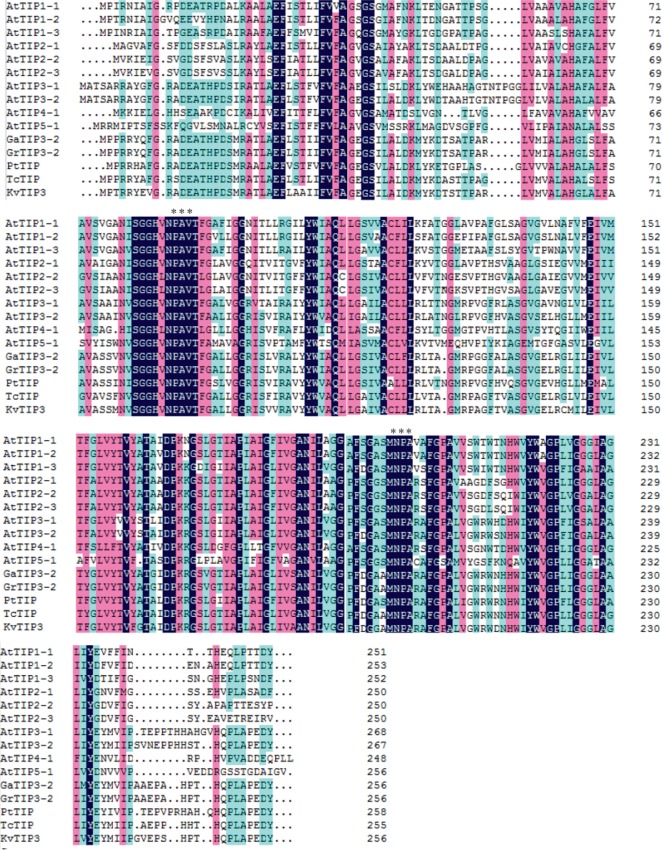
Amino acid sequence alignment of KvTIP3, AtTIP1-1, AtTIP1-2, AtTIP1-3, AtTIP2-1, AtTIP2-2, AtTIP2-3, AtTIP3-1, AtTIP3-2, AtTIP4-1, and AtTIP5-1 from *Arabidopsis thaliana*, GaTIP3-2 from *Gossypium arboreum*, GrTIP3-2 from *Gossypium raimondii*, PtTIP from *Populus trichocarpa*, and TcTIP from *Theobroma cacao*. Different colors in background display the different degrees of conserved sequences.

We further performed bioinformatic analyses. PsortII (http://www.psort.org/) prediction revealed that KvTIP3 protein was located in the secreted pathway with a probability of 94.4%. Further topological prediction with TMHHM (http://www.cbs.dtu.dk/services/TMHMM-2.0/) and TMPred (http://www.ch.embnet.org/software/TMPRED_form.html) demonstrated that KvTIP3 contained six transmembrane helices with its N-terminus and C-terminus located in the cytoplasm (Figures 1(a) and 1(b)). It is well known that the activities of AQPs are regulated by their phosphorylation status. For example, the Snf1-related protein kinase 2.6 (SnRK2.6) was able to phosphorylate a cytosolic PIP2*;*1 peptide at Ser-121 to regulate the response of guard cells to ABA signaling [36]. Similarly, Cys116 and Cys118 have been proved to be the phosphorylation sites in TIP proteins [37]. We also examined the possible phosphorylation sites in KvTIP3 with NetPhos 2.0 (http://www.cbs.dtu.dk/services/NetPhos/) and found that KvTIP3 also contained 7 potential phosphorylation sites, including 4 Ser, 2 Thr, and 1 Tyr (Figure 1(c)). Moreover, we constructed a phylogenetic tree including the typical TIPs in *Arabidopsis* and other closely related organisms. We observed that KvTIP3 was clustered to the *Arabidopsis* AtTIP3 subgroup and was 82% homologous to TIP3-2 in *Gossypium hirsutum*, one of the most closely related organisms to *K. virginica* (Figure 2(d)). In addition, KvTIP3 shared 65%–78% amino acid sequence homology with tonoplast aquaporins from other higher plants (Figure 2). KvTIP3, like TIPs from other higher plant, contained two NPA motifs and formed the cavity of AQPs to act as a selective barrier for the transport of water or small molecules [38].

### 3.3. KvTIP3 Was Induced by Salt Stress in *K. virginica* Seedlings

**Figure 3:**
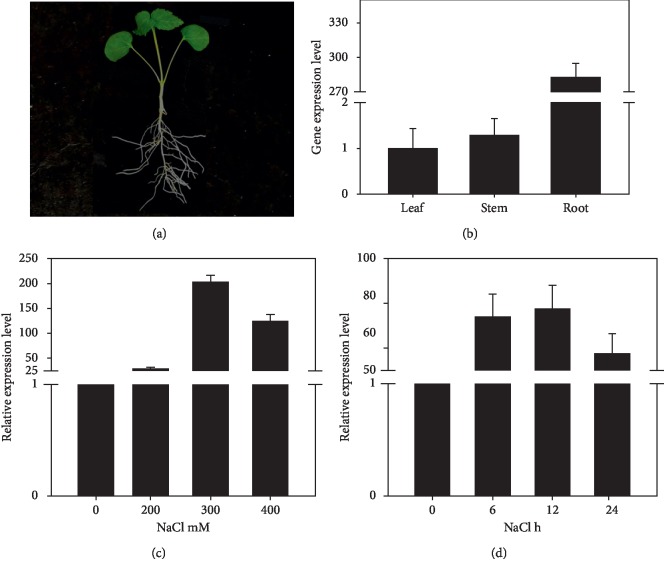
Expression analyses of *KvTIP3*. (a) Phenotype of a two-week-old *K. virginica* seedling used for salt stress or ABA treatment. (b) Expression analysis of *KvTIP3* in the leaves, stems, and roots of two-week-old *K. virginica* seedlings grown under normal growth condition. The expression level in leaves was set as the standard value 1. (c) Expression profiles of *KvTIP3* under different salt stress conditions. Two-week-old *K. virginica* seedlings were treated with 0, 200, 300, and 400 mM NaCl for 24 hours. (d) Expression profiles of *KvTIP3* under salt stress condition. Two-week-old *K. virginica* seedlings were treated with 300 mM NaCl for 0, 6, 12, and 24 hours. The mean values of three biological replicates were used for data analysis. The results of qPCR were normalized with the internal control gene, 18SrRNA. Error bar indicates SD (*n* = 3).

As a first step to know the expression profile of *KvTIP3* in *K. virginica*, we examined its relative expression levels in different tissues of two-week-old seedlings under normal growth condition by qPCR (Figure 3(a)). *KvTIP3* was ubiquitously expressed in various tissues including leaves, stems, and roots, with a predominant expression in the roots (Figure 3(b)). Based on the information extracted from the transcriptomic sequencing database (accession number-GCJL00000000) that *KvTIP3* cDNA fragments were significantly accumulated upon salt stress treatment, we performed qPCR to investigate the transcript levels of *KvTIP3* in *K. virginica* under both normal and salt stress conditions. Two-week-old seedlings were treated with 0, 200, 300, or 400 mM NaCl for 24 h. Although no significant phenotype change was observed after different treatments, salt stress significantly induced the expression of *KvTIP3* in a concentration-dependent manner, with the most significant induction with 300 mM NaCl (Figure 3(c)). When treated with 300 mM NaCl for different time periods, the transcript level of *KvTIP3* increased after 6 h and reached a maximum level at 12 h followed by a gradual decrease at 24 h (Figure 3(d)).

### 3.4. Effects of Various Abiotic Stresses on the Expression of KvTIP3

**Figure 4:**
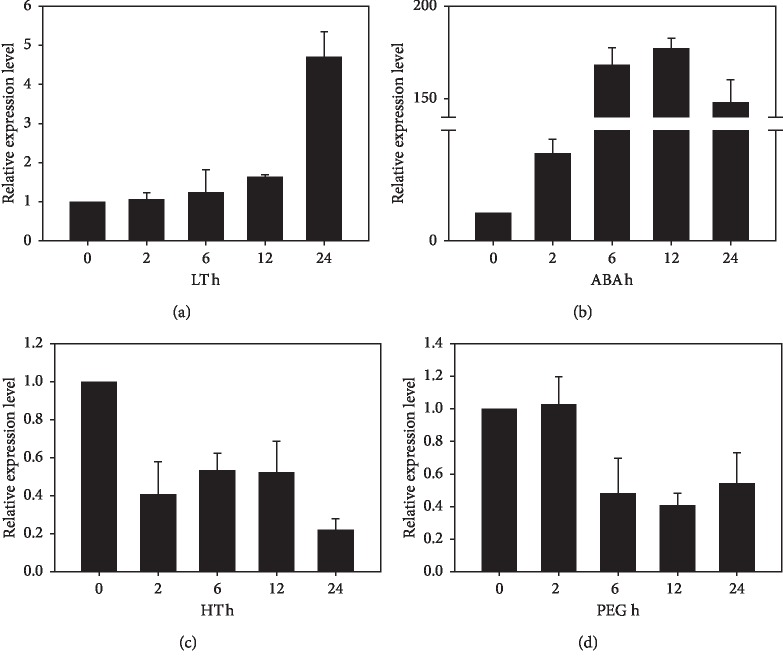
Expression profiles of *KvTIP3* upon different stresses or ABA treatments. (a) Expression profiles of *KvTIP3* upon low temperature treatment. (b) Expression profiles of *KvTIP3* upon ABA treatment. (c) Expression profiles of *KvTIP3* upon high temperature treatment. (d) Expression profiles of *KvTIP3* upon osmotic stress treatment. Error bar indicates SD (*n* = 3).

For the past few years, an increasing number of reports have revealed the important roles of AQPs in abiotic stress responses [39–41]. Thus, we tested the expression of *KvTIP3* under a few abiotic stresses and ABA treatments (Figures 4(a)–4(d)). Similar to the salt stress treatment, both low temperature and ABA treatments upregulated the expression of *KvTIP3*. The expression of *KvTIP3* increased after the seedlings were kept at 4°C for 24 hours (Figure 4(a)), whereas the expression of *KvTIP3* increased within 6 hours after the seedlings were treated with 100 *μ*M ABA (Figure 4(b)). Different from these observations, the expression of *KvTIP3* was downregulated when the seedlings were kept at 42°C or treated with 15% PEG (Figures 4(c) and 4(d)). All these results suggest that *KvTIP3* is an abiotic stress and ABA inducible or responsive gene.

## 4. Discussion

Previous studies have demonstrated that AQPs play important roles in plant growth, development, and in plant resistance to adverse environmental stresses. In Tulip (*Tulipa gesneriana*), both TgTIP1;1 and TgTIP1;2 were involved in petal development by regulating the absorption and transport of water [42]. Under osmotic or salt stress condition, AQPs in cucumber seedlings were able to regulate root and leaf hydraulic properties to respond to the environmental stimulus [41]. In this work, we isolated a novel AQP encoding gene *KvTIP3* from *K*. *virginica*. Acting as multifunctional membrane channels, AQPs have very highly conserved structure [7, 18]. Sequence analyses of KvTIP3 revealed that it was one of the homologs of AQPs. The molecular weights of the reported AQPs ranged from 23 to 31 kDa (243–302 amino acid residues). We found that *KvTIP3* gene encoded a 27 kDa AQP that consisted of 256 amino acid residues.

Similar to the AQPs from other plant species, KvTIP3 also contained six membrane-spanning *α*-helices, as predicted with both TMHHM and TMPred software programs (Figures 1(a) and 1(b)). The water channel activities of AQPs were activated and deactivated via the phosphorylation and dephosphorylation of their phosphorylation sites [43]. The Snf1-related protein kinase 2.6 (SnRK2.6) was reported to be able to phosphorylate AQPs [36]. The C-termini of several PIPs were also discovered to be phosphorylated by a sugar-induced receptor kinase [44]. We found that KvTIP3 contained 7 potential phosphorylation sites, implying that it may also play its functions via phosphorylation mechanism (Figure 1(c)). Further analyses indicated that KvTIP3 was homologous to and shared amino acid sequence homology with other plant AQPs (Figure 1(d)). It is well known that the conserved NPA motif in AQPs functions as a selective filter for the substrate specificity [44]. Sequence analysis showed that KvTIP3 contained two NPA motifs, indicating that the KvTIP3 was a typical AQP (Figure 2).

The expression pattern of AQPs may affect their biological functions in the growth, development, and response to environmental stress of plants. We observed that the expression level of *KvTIP3* in roots was over 270-folds higher than in leaves and stems (Figure 3(b)). Similar expression pattern of AQPs was also observed in *Arabidopsis* [45], rice [46], grape [47], and barley [48]. In addition, the *KvLEA* gene which we reported previously also showed a similar expression pattern, indicating that these genes play important roles in roots of plants to improve salt resistance [30]. Staying the same with our transcriptome analysis, *KvTIP3* could be induced obviously by NaCl treatment [28]. However, different from *KvLEA* gene [30], 300 mM NaCl led to the most drastic accumulation to *KvTIP3* rather than 400 mM (Figure 3(c)). As for the different time of duration treatments, *KvTIP3* gave the same expression profiles with *KvLEA*, proving that both *KvTIP3* and *KvLEA* were salt stress responsive genes of *K. virginica* (Figure 3(d)).

We also examined the expression levels of *KvTIP3* in response to different abiotic stresses and ABA treatment. *KvTIP3* was upregulated by low temperature and ABA but downregulated by high temperature and osmotic stress (Figures 4(a)–4(d)). This is consistent with previous studies that the responses of AQPs under adverse environment were diverse [49]. The responses of *KvTIP3* under high temperature and osmotic stress were not obvious (Figures 4(c) and 4(d)). Therefore, we speculated that *KvTIP3* may not be involved in high temperature response. However, TIPs were reported to take part in the response to osmotic stress [12]. In this research, the result showed that there should be other *KvTIPs* that participate in osmotic stress response in *K. virginica* rather than *KvTIP3*. Besides, ABA treatment gave rise to the most drastic accumulation to *KvTIP3* among these treatments (Figure 4(b)). For instance, the expression of *KvTIP3* reached as much as 180-fold after 6h ABA treatment. And the expression of *KvTIP3* increased rapidly as soon as after 2 h ABA treatment; hence, we speculated that *KvTIP3* may be involved in ABA-mediated stress responses in *K. virginica*. Actually, the intersections between ABA and AQPs have already been reported in other species. In *Arabidopsis*, AQPs functioned in ABA-triggered stomatal closure [36], and the vacuolar aquaporins were able to maintain seed longevity under the control of ABA [50]. In addition, ABA also could influence the phosphorylation of PIPs to regulate their activities [51]. Therefore, the exact mechanisms between *KvTIP3* and ABA deserve further investigation in the following research.

## 5. Conclusions

In this report, we characterized a novel tonoplast intrinsic protein gene *KvTIP3* from *K. virginica*. Bioinformatic analyses revealed that KvTIP3 is a typical AQP with conserved transmembrane helices and NPA motifs. Gene expression profiles displayed that it was predominately expressed in roots and was responsive to various abiotic stresses. Our study on *KvTIP3* will enrich the gene resources and lay foundation for further study on AQPs. *KvTIP3* could be used as a potential candidate gene for the improvement of plants resistant to various abiotic stresses in the future.

## Data Availability

The data used to support the findings of this study are included within the article.
